# LncRNA SNHG15 modulates gastric cancer tumorigenesis by impairing miR-506-5p expression

**DOI:** 10.1042/BSR20204177

**Published:** 2021-07-28

**Authors:** Zhiping Chen, Tianyu Zhong, Tao Li, Jinghua Zhong, Yang Tang, Zhanyu Liu, Baodian Ling, Lanfeng Wang

**Affiliations:** 1 Department of Laboratory Medicine, First Affiliated Hospital of Gannan Medical University, Jiangxi Province, China; 2 Department of Oncology, First Affiliated Hospital of Gannan Medical University, Jiangxi Province, China; 3 Department of Traditional Chinese Medicine, First Affiliated Hospital of Gannan Medical University, Jiangxi Province, China; 4 Department of Nephrology, First Affiliated Hospital of Gannan Medical University, Jiangxi Province, China

**Keywords:** Apoptosis, Gastric cancer, LncRNA SNHG15, MicroRNA-506-5p, Proliferation

## Abstract

The gastric cancer (GC) patients commonly have a poor prognosis due to its invasiveness and distant metastasis. Growing evidence proved that aberrant long non-coding RNAs (lncRNAs) expression contributes to tumor development and progression. LncRNA SNHG15 has been reported to be involved in many different kinds of cancer, while its role in GC remains unclear. In the present study, we found that SNHG15 was up-regulated in GC tissues and cell lines. Silencing SNHG15 suppressed proliferation migration, invasion and promoted apoptosis of AGS cells. More importantly, microRNA-506-5p (miR-506-5p) was predicted as a direct target of SNHG15 by binding its 3′-UTR and further verified using luciferase reporter assay. Meanwhile, the results of rescue experiments revealed that knockdown of miR-506-5p expression reversed the functional effects of SNHG15 silenced cell proliferation, migration, invasion and apoptosis. In conclusion, our findings revealed that SNHG15 executed oncogenic properties in GC progression through targeting miR-506-5p, which might provide a novel target for the GC treatment.

## Introduction

Gastric cancer (GC) is the most common malignant human tumor which has high morbidity and mortality. There were over 1 million patients diagnosed with GC (5.7% of all cancer diagnoses) and the deaths were over 780000 (8.2% of all cancer-associated mortalities) in 2018 [1,2]. With the development of advanced medical facility in therapeutic strategies, most patients with GC have been diagnosed at advanced stage [3–5]. At present, the treatment effect of patients with advanced patients is very poor due to the influence of tumor metastasis and drug resistance. Therefore, an intensive understanding of the pathogenesis of GC will be helpful in improving diagnosis and therapy for GC patients.

Long non-coding RNAs (lncRNAs) are a type of RNA which are longer than 200 nucleotides in length [6], and mainly act as molecular sponges, preventing microRNAs (miRNAs) from binding to mRNA targets and antagonizing their functions [7–10]. Increasing evidence have reported that lncRNAs possess extensive regulatory roles in the occurrence and development of diseases (especially cancers). Number of studies demonstrated that lncRNAs participated in multiple biological processes, including cell proliferation, invasion, apoptosis and differentiation [11–14].

Mounting evidence has proven that lncRNAs serve as important regulators in the development and progression of GC. In recent years, multiple lncRNAs have been shown to be dysregulated in GC, such as HOXA11-AS [15], AC130710 [16], MACC1-AS1 [17] and PVT1 [18]. A study has found that lncRNA H19 could enhance GC carcinogenesis and metastasis [19]. In addition, lncRNA LINC00483 promotes GC development through regulating miR-490-3p/MAPK1 axis [20]. LncRNA SNHG15 has been previously shown to accelerate cell proliferation and migration of lung cancer [21], osteosarcoma [22], nasopharyngeal carcinoma [23] and other tumors. Although SNHG15 has been investigated in other cancers, the role of SNHG15 and the molecular mechanisms in GC remains unclear. In the present study, we aimed to shed some light on the biological role and investigate the latent mechanism of SNHG15 on the occurrence and development of GC.

## Materials and methods

### Clinical tissues

Thirty pairs of freshly dissected GC tissues and adjacent normal tissues between June 2018 and December 2019, were acquired from patients who underwent surgery at the First Affiliated Hospital of Gannan Medical University. Certainly, all patients did not receive any chemotherapy before sampling and signed with informed consent. Our project was approved by the Ethics Committee of the First Affiliated Hospital of Gannan Medical University. Tissues were immediately stored at −80°C before use.

### Cell culture

Three GC cell lines (AGS, MNK-45, SNU-1) and human gastric epithelial cell line, GES-1, were purchased from American Type Culture Collection (ATCC, Rockville, MD, U.S.A.). Cells were cultivated in Dulbecco’s Modified Eagle’s Medium (DMEM; Invitrogen, Carlsbad, U.S.A.) supplemented with 10% fetal bovine serum (FBS) at 37°C in a humidified atmosphere comprising 5% CO_2_.

### Cell transfection

AGS cells (1 × 10^5^) were seeded into six-well plates and cultured to 70–80% confluence. Specific siRNAs against SNHG15 (si-SNHG15), si-NC, microRNA-506-5p (miR-506-5p) inhibitor or inhibitor NC were constructed by Genechem (Shanghai, China) and transfected into cells with Lipofectamine 2000 (Thermo Fisher Scientific, Waltham, U.S.A.). Transfected cells were subjected to quantitative real-time PCR (qRT-PCR) analysis to detect the transfection efficacy.

### RNA extraction and qRT-PCR analysis

TRIzol reagent (Takara, Japan) was applied to isolate the total RNA from tissues and cells. RNA was reverse-transcribed into cDNA using PrimeScript RT reagent kit (Takara, Japan). qRT-PCR was carried out by SYBR Green qPCR Master Mix (Thermo Fisher Scientific). qRT-PCR amplification conditions were as follows: 95°C for 10 min, followed by 43 cycles each at 95°C for 10 s, 60°C for 30 s, 72°C for 30 s. GAPDH and U6 were used as the internal reference and 2^−ΔΔ*C*_T_^ method was applied to calculate the gene expression.

### Western blot analysis

Total proteins were extracted from cells using RIPA buffer (Beyotime, China). BCA assay kit (Beyotime, Shanghai, China) was applied to detect total protein concentration. Protein was separated on a 10% SDS/PAGE gel and then transferred on to PVDF membranes (Keygen, Nanjing, China) following blocking with 5% non-fat milk for 1 h at 37°C. Subsequently, membranes were probed with primary antibodies overnight at 4°C, washed with TBS with Tween-20 and hatched with secondary antibodies at room temperature for 2 h. The expression levels of protein were visualized by an ECL detection kit (Thermo Scientific, U.S.A.).

### Cell counting kit-8 assay

We assessed cell proliferation using cell counting kit-8 (CCK-8) assay. In brief, cells (1 × 10^5^) were seeded on to 96-well plates, cultured for another 0, 24, 48 and 72 h, and then processed with 10 μl of CCK-8 reagent (Gibco, U.S.A.). At last, the optical density (OD) was measured at 450 nm using a microplate reader (BMG Labtech, Germany). These tests were carried out in triplicate.

### EdU analysis

5-ethynyl-2′-deoxyuridine (EdU) assay kit (Thermo Fisher Scientific) was applied to explore cell proliferation. Cells (1 × 10^5^) were maintained in six-well plates and 100 μl of EdU was added for 2 h. Afterwards, cells were treated with 4% paraformaldehyde following addition of 0.5% Triton X-100. Finally, cells were stained with anti-EdU solution. EdU-positive cells were analyzed under a fluorescence microscopy (Olympus, Tokyo, Japan).

### Flow cytometry analysis

Cells (1 × 10^5^) were centrifuged and stained using Annexin V-fluorescein isothiocyanate (FITC) and propidium iodide (PI) kits (Beyotime, China) for detecting cell apoptosis and quantified using flow cytometry on a Beckman Coulter flow cytometer (Becton Dickinson, U.S.A.). The data were analyzed by FlowJo v10 software (Tree Star, Inc.).

### Transwell chamber analysis

We used a Transwell Chamber (8.0 µm; Millipore, Billerica, MA, U.S.A.) to explore the cell migration and invasion abilities. Cells were seeded at 24-well upper uncoated chambers (BD Biosciences, CA, U.S.A.) with serum-free medium for migration analysis and the upper chamber loaded with matrigel (BD Biosciences) for invasion analysis. After 48-h incubation, the cells which did not migrate and invade the lower chamber were wiped with a cotton swab. Cells were fixed with 4% paraformaldehyde, followed by staining with 1% Crystal Violet. The number of migrated or invaded cells was counted under a light microscope (Olympus Corporation, Tokyo, Japan) (×200 magnification) in five random fields.

### Luciferase reporter assay

We predicted the SNHG15-binding sites of miR-506-5p with bioinformatics tools. Firstly, the wildtype (WT) or mutant type (Mut) of SNHG15 3′UTR was subcloned into pmirGLO dual-luciferase vector. Then, they were co-transfected into cells with miR-506-5p mimic or NC mimic by using Lipofectamine 2000 (Invitrogen, U.S.A.). At 48 h of transfection, the relative activity of luciferase was detected by Dual-Luciferase Reporter Assay System (Promega, Madison, WI, U.S.A.). The *Renilla* luciferase activity was normalized.

### Statistical analysis

The tests were conducted for three times independently and data were presented as mean ± SD. Statistical analyses were performed using SPSS v19.0 software (IBM Corp.). Two groups of experiments were conducted using Student’s *t* test. One-way ANOVA, followed by Tukey’s post hoc test was applied to analyze the differences among multiple groups. *P*<0.05 was considered as statistical significance.

## Results

### SNHG15 expression is up-regulated in GC tissues and cell lines

**Figure 1:**
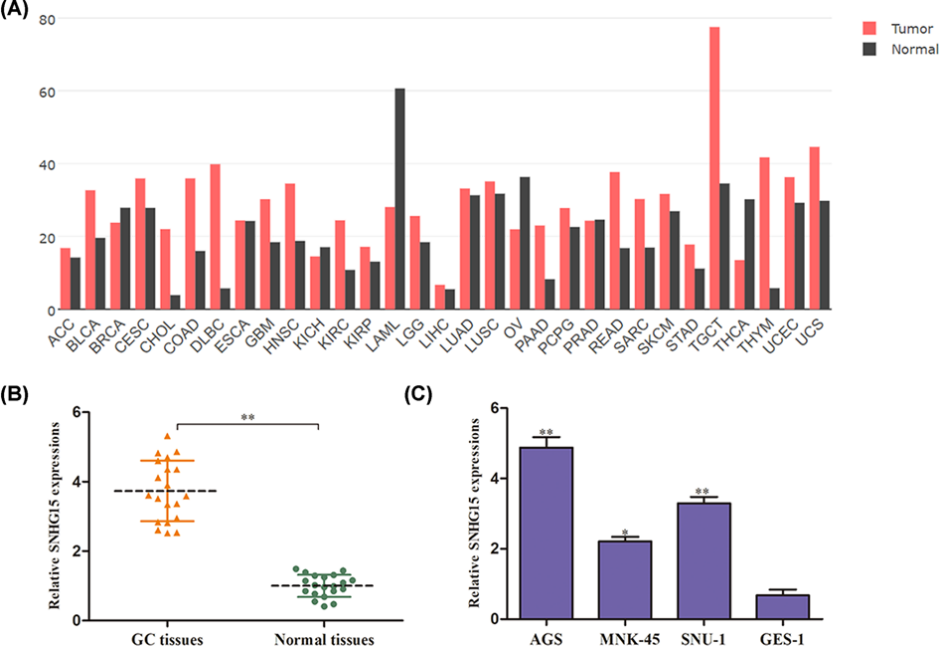
SNHG15 expression is up-regulated in GC tissues and cell lines (**A**) GEPIA database of the expression of SNHG15 in GC tissues. (**B,C**) Expression level of SNHG15 in GC tissues and cell lines. The experiment was repeated in triplicate. *P<0.05, **P<0.01 *vs*. normal tissues or GES-1 cell lines.

To figure out the role of SNHG15 in HCC, we explored its expression by searching the Cancer Genome Atlas (TCGA) and Gene Expression Profiling Interactive Analysis (GEPIA) databases. The GC tissues expressed higher levels of SNHG15 than the normal tissues (Figure 1A). Next, to explore the roles of SNHG15 in GC, qRT-PCR was conducted to assess SNHG15 expression in GC tissues and cell lines. The result indicated that SNHG15 expression pattern was dramatically overexpressed in GC tissues compared with corresponding normal tissues (Figure 1B). Likewise, SNHG15 was notably up-regulated in GC cell lines (AGS, MNK-45, SNU-1), especially in AGS cells accompany with the normal epithelial cells (GES-1) (Figure 1C). Based on these results, we concluded that SNHG15 might be a prognostic biomarker in the progression of GC.

### SNHG15 knockdown inhibits GC cell proliferation

**Figure 2:**
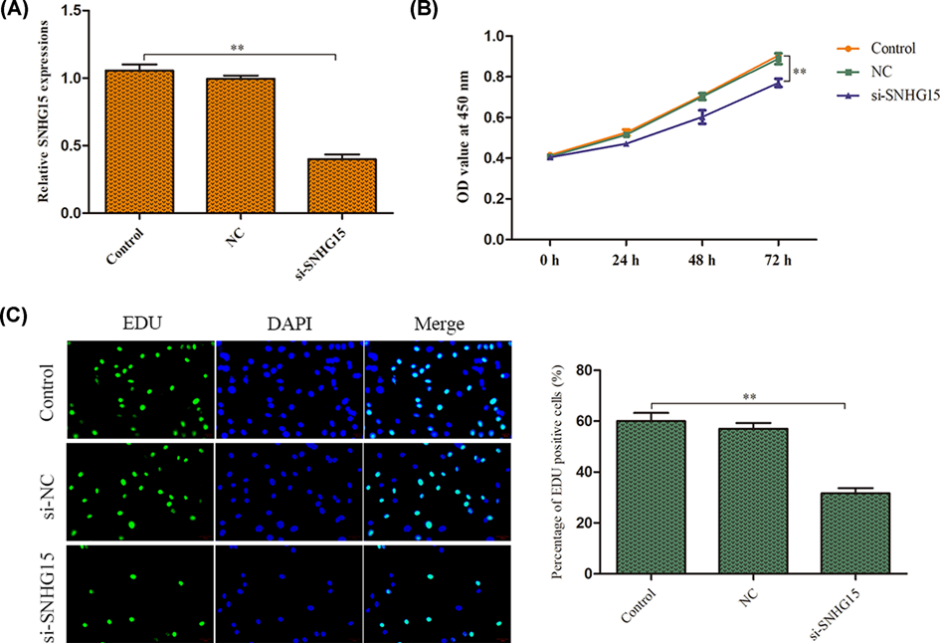
SNHG15 knockdown inhibits GC cell proliferation (**A**) Transfection efficiency was verified by qRT-PCR analysis. (**B**) Cell viability was detected by CCK-8 assay. (**C**) Cell proliferation was estimated by EdU assay. The experiment was repeated in triplicate. **P<0.01 *vs*. Control group.

In order to explore the biological role of SNHG15 in GC, loss-of-function assays were implemented. Si-SNHG15 was transfected into AGS cells for SNHG15 knockdown and the efficacy of transfection was verified by qRT-PCR analysis. The data showed that SNHG15 was down-regulated following si-SNHG15 transfection (Figure 2A). To further explore the roles of SNHG15 in GC cell proliferation, CCK-8 assay delineated that SNHG15 knockdown inhibited cell viability, compared with the control group (Figure 2B). Consistently, EdU analysis indicated that down-regulated SNHG15 expression suppressed the proliferative ability of AGS cells when compared with control group (Figure 2C).

### SNHG15 knockdown accelerates cell apoptosis

**Figure 3:**
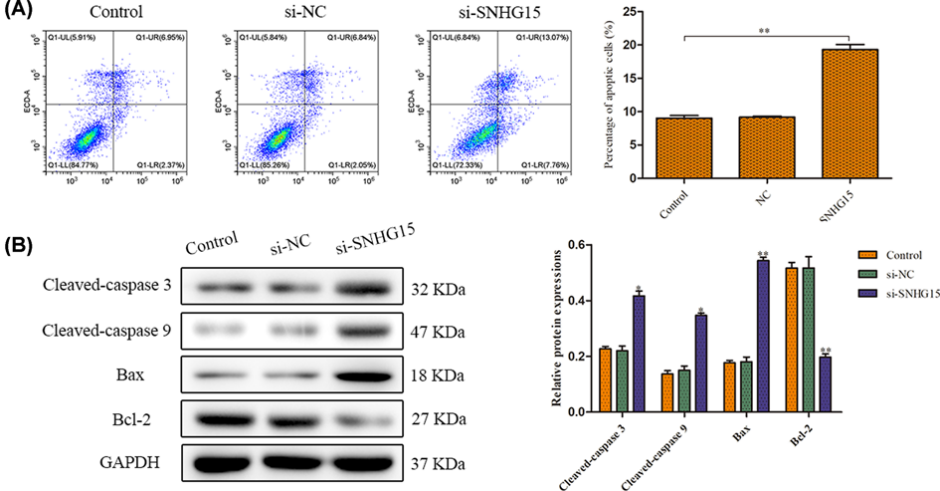
SNHG15 knockdown accelerates cell apoptosis (**A**) Cell apoptosis was detected by the flow cytometry. (**B**) Expression levels of apoptosis-related proteins were detected by the Western blot assay. The experiment was repeated in triplicate. *P<0.05, **P<0.01 *vs*. Control group.

The effect of SNHG15 expression on cell apoptosis was evaluated using flow cytometry analysis. The results expounded that down-regulation of SNHG15 expression evidently increased augmentation of apoptotic cells (Figure 3A). Furthermore, pro-apoptosis protein, Bax expression was increased after transfection with si-SNHG15. In contrast, the expression of Bcl-2, an inhibitor for cell apoptosis was abolished in si-SNHG15 group, when compared with control group (Figure 3B).

### Down-regulation of SNHG15 impedes migration and invasion of GC cells

**Figure 4:**
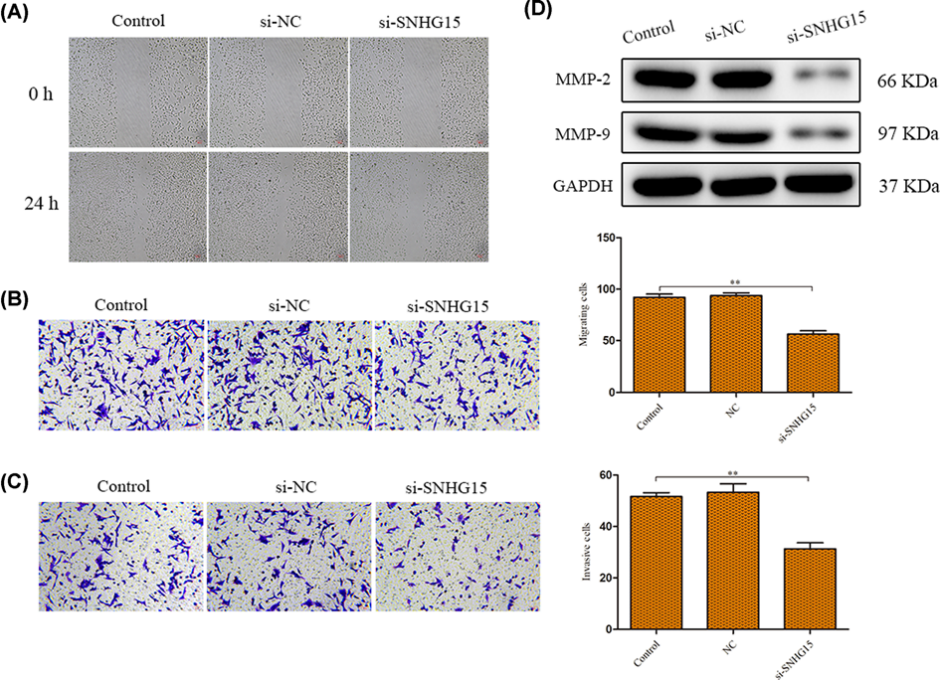
Down-regulation of SNHG15 impedes migration and invasion of GC cells (**A–C**) Cell migration and invasion were detected by the wound healing and transwell chamber assays. (**D**) The protein expression levels of migration- and invasion-related proteins in AGS cells. The experiment was repeated in triplicate. **P<0.01 *vs*. Control group.

Subsequently, we explored the effects of SNHG15 on cell migration and invasion abilities by using wound healing and transwell assays. The results showed that knockdown of SNHG15 expression effectively suppressed the migration ability of cells (Figure 4,B). Similarly, when compared with control group, the invasion ability of AGS cells was alleviated by si-SNHG15 transfection (Figure 4C). More importantly, migration- and invasion-related protein expressions (MMP-2 and MMP-9) were detected. Western blot analysis elucidated that silencing the SNHG15 expression resulted in the reduction of MMP-2 and MMP-9 expression levels (Figure 4D).

### SNHG15 sponges miR-506-5p in GC cells

**Figure 5:**
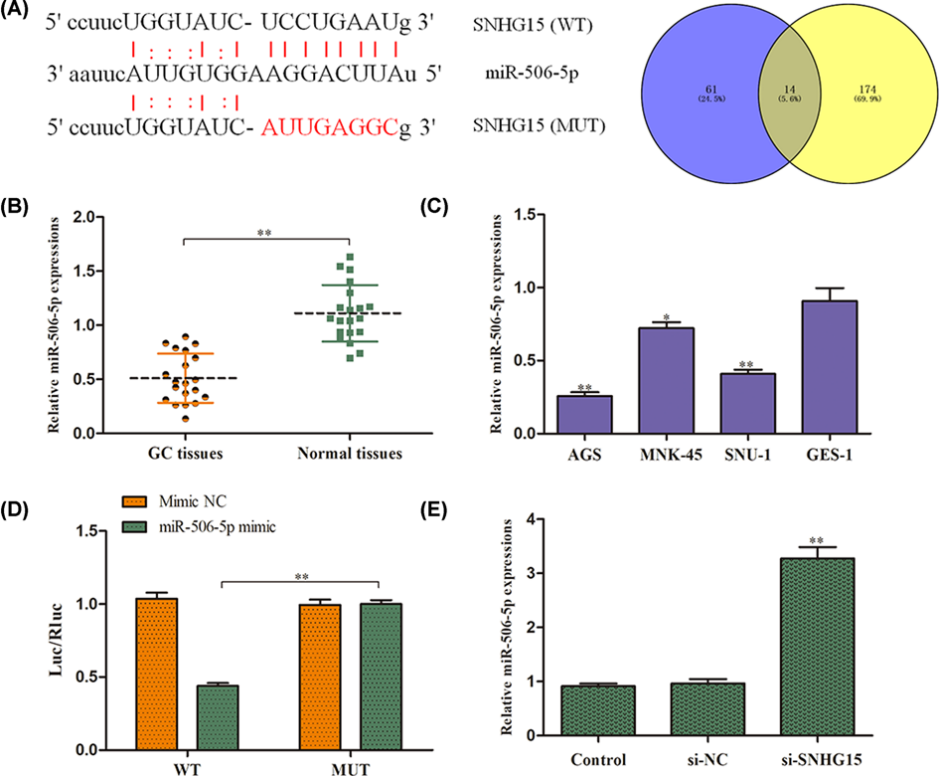
SNHG15 sponges miR-506-5p in GC cells (**A**) The miR-506-5p binding sites in the 3′UTR of SNHG15 as predicted by StarBase. (**B,C**) qRT-PCR analysis of miR-506-5p expression levels in GC tissues and cell lines. (**D**) Luciferase reporter assay was performed to confirm the interaction between SNHG15 and miR-506-5p. (**E**) The expression of miR-506-5p was examined by qRT-PCR assay. The experiment was repeated in triplicate. **P<0.01 *vs*. Normal tissues, GES-1 cell lines, mimic NC or Control group.

In order to probe the potential regulatory molecular mechanism through which SNHG15 affected GC progression, we proceeded bioinformatics analysis by StarBase database and found that SNHG15 was uncovered to possess speculated binding sites with miR-506-5p (Figure 5A). Subsequently, the expression levels of miR-506-5p were measured by qRT-PCR analysis in GC tissues and cells. We noted that miR-506-5p was lowly expressed in the GC tissues (Figure 5B). Consistently, miR-506-5p expression was also markedly decreased in GC cell lines (Figure 5C). To determine the interaction of SNHG15 and miR-506-5p, we performed dual luciferase reporter analysis and indicated that luciferase activity have a significant reduction of SNHG15-WT when co-transfected with miR-506-5p mimics in AGS cells. However, there was no obvious alteration in luciferase activity of SNHG15-Mut (Figure 5D). Accordingly, down-regulation of SNHG15 distinctly increased the expression miR-506-5p in AGS cells (Figure 5E).

### SNHG15 accelerates CRC progression via targeting miR-506-5p

**Figure 6:**
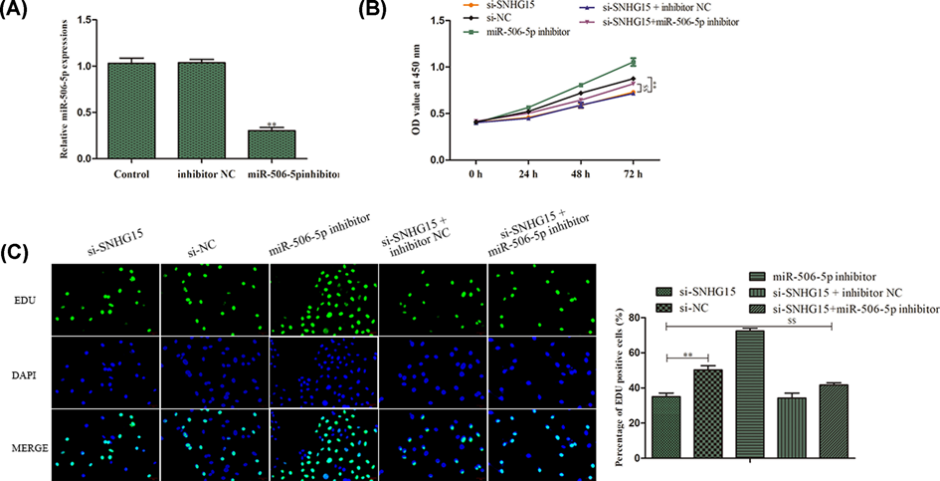
SNHG15 accelerates CRC progression via targeting miR-506-5p (**A**) The transfection efficiency of miR-506-5p was verified by qRT-PCR. (**B,C**) CCK-8 and EdU assays were carried out to assess the proliferative capacity of AGS cells. The experiment was repeated in triplicate. **P<0.01 *vs*. si-NC group. ^$$^*P*<0.01 *vs*. si-SNHG15 group.

**Figure 7:**
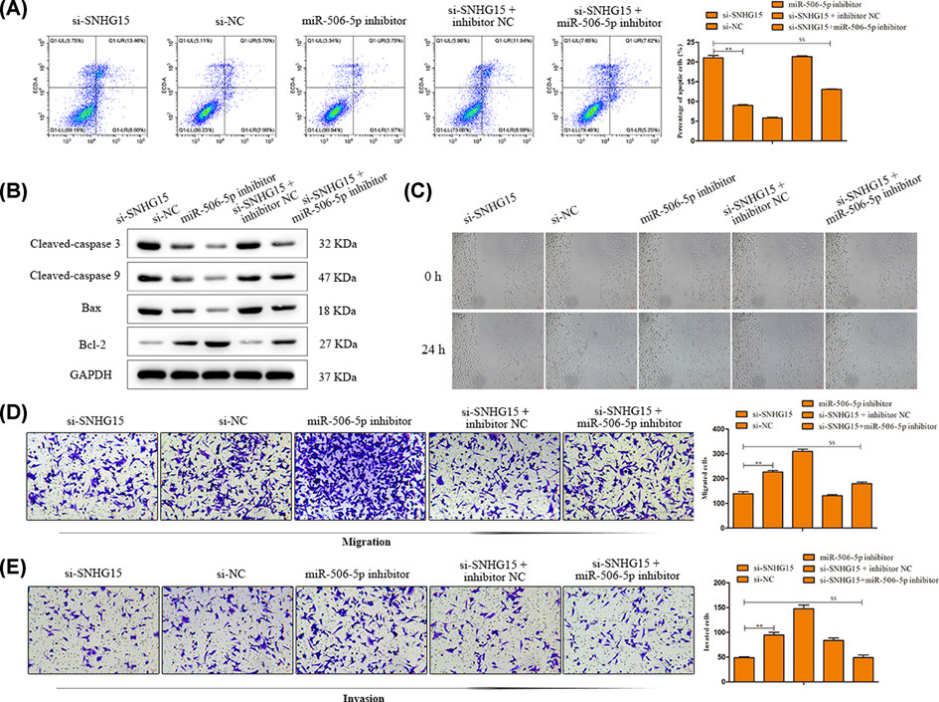
SNHG15 accelerates CRC progression via targeting miR-506-5p (**A**) Cell apoptosis was detected by flow cytometry. (**B**) The apoptosis-related protein expressions were analyzed. (**C–E**) Cell migration and invasion were detected by the wound healing and transwell chamber assays. The experiment was repeated in triplicate. **P<0.01 vs. si-NC group. ^$$^*P*<0.01 vs. si-SNHG15 group.

Based on the above results, rescue assays were conducted to demonstrate whether SNHG15 elicited its performance through targeting miR-506-5p in GC. At first, RT-PCR assay was applied to certify the effectiveness of transfection for miR-506-5p and demonstrated that transfected miR-506-5p inhibitor gave rise to the decline of miR-506-5p expression compared with NC group (*P<*0.01; Figure 6A). Next, CCK-8 and EdU assays depicted that SNHG15 knockdown-induced reduction in cell proliferation ability of AGS cells, whereas the impact of SNHG15 was subsequently recovered by miR-506-5p inhibitor (*P*<0.05, Figure 6B,C). Furthermore, flow cytometry analysis indicated that the apoptosis was evidently facilitated when SNHG15 expression was inhibited, while miR-506-5p inhibitor relieved these phenomena (Figure 7A). In addition, the apoptosis-related protein expressions were analyzed using Western blot assay. As shown in Figure 7B, the effect of SNHG15 depletion on the expression levels of cleaved caspase 3, cleaved caspase 9, Bcl-2 and Bax was abrogated by inhibition of miR-506-5p. Furthermore, similar results were observed in wound healing, transwell migration and invasion assays, the inhibitory influences of SNHG15 knockdown on cell migration and invasion were abolished by inhibition of miR-506-5p (Figure 7C–E).

## Discussion

Recently, the research on lncRNAs has received more attention. More and more studies are showing that lncRNAs may play important roles in the progression of human cancers, including GC [24–27]. For example, lncRNA NLIPMT overexpression inhibits cell migration by down-regulating TGF-β1 in colorectal cancer [28]. Silencing of lncRNA HEIH inhibits liver cancer cell growth and metastasis through miR-199a-3p/mTOR axis [29]. Hui et al. have found that FEZF1-AS1 modulates cancer stem cell properties through miR-363-3p/HMGA2 axis in GC [30]. LncRNA DCST1-AS1 has proved that it could regulate cell proliferation and apoptosis in GC by targeting miR-605-3p [31]. Therefore, dissecting the role of lncRNAs in GC progression is important for the identification of GC clinical treatment.

In our study, we evaluated the expression levels of SNHG15 in human GC tissues and cell lines, and found that it was up-regulated both in tissues and cells. Additionally, the functional analysis unveiled that SNHG15 knockdown executed tumor-suppressing properties in the deterioration of GC via inhibiting cell proliferation, migration, invasion and facilitating apoptosis.

The importance of lncRNAs in human disease may be associated with their ability to impact cellular functions through various mechanisms. A myriad of investigations have revealed that lncRNAs are able to act as molecular sponges of miRNAs [32–34]. miRNAs are small, approximately 22-nucleotide, noncoding RNAs which work as oncogenic or tumor suppressor genes in diverse malignant tumors, including GC [16,17]. For instance, miRNA-222 accelerates colorectal cancer cell migration and invasion by targeting MST3 [34]. Ran et al. found that miR-194 inhibits liver cancer stem cell expansion by regulating RAC1 pathway [35]. In addition, lncRNA LOXL1-AS1 facilitates non-small-cell lung cancer cell proliferation by targeting miR-324-3p [36]. To investigate the miRNA-related functions of SNHG15 in gastric pathogenesis, we chose miR-506-5p as a model miRNA for further studies. More importantly, miR-506-5p was identified as a functional target gene of SNHG15 through bioinformatics analysis and dual luciferase reporter analysis. Finally, we demonstrated that miR-506-5p inhibition could rescue the inhibition effect of SNHG15 down-regulation on GC progression. Consistent with the above findings, our findings also suggest that SNHG15 knockdown inhibits cell proliferation and invasion by targeting miR-506-5p expression.

In the present study, our findings revealed that SNHG15 was increased in GC tissues and cells. Functionally, knockdown of SNHG15 inhibits the proliferation, migration, invasion and accelerates apoptosis of GC. In summary, our study indicated that SNHG15 deteriorated GC progression at least in part by inhibiting miR-506-5p expression levels. Our findings imply that SNHG15 functions as an oncogene and may serve as a novel target for the diagnosis and treatment of GC.

## Supplementary Material

Supplementary Files

## Data Availability

All datasets generated for the present study are included in the manuscript and/or the supplementary files.

## Abbreviations

Bax: BCL2 associated X
Bcl-2: B-cell lymphoma-2
CCK-8: cell counting kit-8
CRC: colorectal carcinoma
EdU: 5-ethynyl-2′-deoxyuridine
GC: gastric cancer
lncRNA: long non-coding RNA
miRNA: microRNA
miR-506-5p: microRNA-506-5p
MMP: matrix metalloproteinase
qRT-PCR: quantitative real-time PCR
SNHG15: LncRNA SNHG15
