# Supplementary material for: LncRNA SNHG15 modulates gastric cancer tumorigenesis by impairing miR-506-5p expression
Source: Biosci Rep. 2021 Jul 28;41(7):BSR20204177. doi: 10.1042/BSR20204177 (PMC8319491; doi:10.1042/BSR20204177)
Supplement: Supplementary Files [file BSR-2020-4177_supp.pdf]

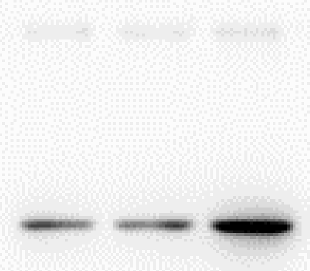

Figure 3B-Bax (18kD)

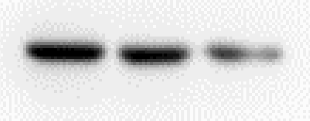

Figure 3B-Bcl-2 (27kD)

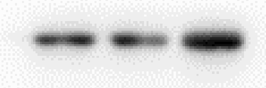

Figure 3B-Cleaved-caspase-3 (32kD)

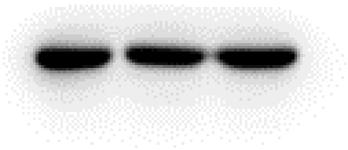

Figure 3B-GAPDH (37kD)

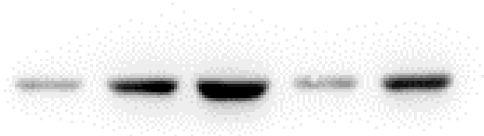

Figure 7B-Bcl-2 (27kD)

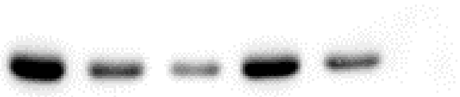

Figure 7B-Cleaved-caspsase-3 (32kD)

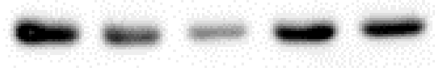

Figure 7B-Cleaved-caspsase-9 (47 kD)

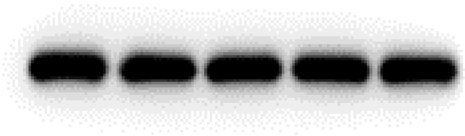

Figure 7B-GAPDH (37 kD)

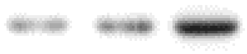

Figure 3B-Cleaved-caspase-9 (47kD)

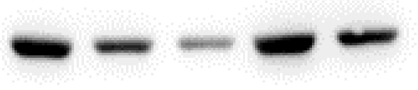

Figure 7B-Bax (18kD)
